# Effect of Smoking on Coronary Artery Plaques in Type 2 Diabetes Mellitus: Evaluation With Coronary Computed Tomography Angiography

**DOI:** 10.3389/fendo.2021.750773

**Published:** 2021-11-03

**Authors:** Yu Jiang, Tong Pang, Rui Shi, Wen-lei Qian, Wei-feng Yan, Yuan Li, Zhi-gang Yang

**Affiliations:** Department of Radiology, West China Hospital, Sichuan University, 37# Guo Xue Xiang, Chengdu, China

**Keywords:** type 2 diabetes mellitus, smoking, coronary computed tomographic angiography, coronary artery plaques, atherosclerosis

## Abstract

**Background:**

The effect of smoking on coronary artery plaques examined by coronary computed tomography angiography (CCTA) in type 2 diabetes mellitus (DM) patients is not fully understood. This study explored the effect of smoking on coronary artery plaques by comparing the characteristics of plaques between diabetes patients with and without a smoking history and among those with different smoking durations.

**Materials and Methods:**

In total, 1058 DM patients found to have coronary plaques on CCTA were categorized into the smoker (n=448) and nonsmoker groups (n=610). Smokers were stratified by smoking duration [≤20 years (n=115), 20~40 years (n=233) and >40 years (n=100)]. The plaque types, luminal stenosis [obstructive (<50%) or nonobstructive (≥50%) stenosis], segment involvement score (SIS), and segment stenosis score (SSS) of the CCTA data were compared among groups.

**Results:**

Compared to nonsmokers, smokers demonstrated increased odds ratios (ORs) of any noncalcified plaques (OR=1.423; P=0.014), obstructive plaques (OR=1.884; P<0.001), multivessel disease (OR=1.491; P=0.020), SIS≥4 (OR=1.662; P<0.001), and SSS≥7 (OR=1.562; P=0.001). Compared to diabetes patients with a smoking duration ≤20 years, those with a smoking duration of 20~40 years and >40 years had higher OR of any mixed plaques (OR=2.623 and 3.052, respectively; Ps<0.001), obstructive plaques (OR=2.004 and 2.098; P=0.003 and 0.008, respectively), multivessel disease (OR=3.171 and 3.784; P<0.001 and P=0.001, respectively), and SSS≥7 (OR=1.605 and 1.950; P=0.044 and 0.020, respectively). Diabetes with a smoking duration >40 years had a higher OR of SIS≥4 (OR=1.916, P=0.034).

**Conclusion:**

Smoking is independently associated with the presence of noncalcified, obstructive, and more extensive coronary artery plaques in diabetes patients, and a longer smoking duration is significantly associated with a higher risk of mixed, obstructive, and more extensive plaques.

## Introduction

Diabetes seriously threatens global health, and the number of adults with diabetes has more than tripled over the past 20 years (1). For type 2 diabetes mellitus (DM) patients, cardiovascular disease, including coronary artery disease, is a major cause of morbidity and mortality. As one of the strongest predictors of the risk of death among patients with DM, smoking has also been proven to be strongly associated with a long-term risk of coronary artery disease (2, 3). Approximately 20% of DM patients currently use tobacco worldwide (4). Unlike many other risk factors for atherosclerosis, smoking is a modifiable factor for adverse health consequences and cardiovascular outcomes in DM (5–7). Thus, identifying the relationship of smoking with coronary artery disease in DM patients can be beneficial for better clinical practice recommendations.

Coronary computed tomographic angiography (CCTA), a noninvasive diagnostic technique, has played an important role in detecting the presence of coronary artery plaques and assessing the severity of coronary artery stenosis for a long time, and treatment decision-making in coronary artery disease based solely on CCTA is potentially feasible because of its strong agreement with decisions derived from conventional coronary angiography (8). Previous CCTA findings demonstrate a heavy coronary plaque burden in DM patients (9, 10). Cigarette smoking has been proven to be associated with the presence, extent, and severity of atherosclerosis on CCTA (11), while the contribution of smoking to the development of coronary artery plaques detected by CCTA in DM patients is not fully understood. This study aimed to explore the effect of smoking on coronary artery plaque by comparing the characteristics of coronary artery plaque between DM patients with and without a history of smoking and among DM patients with different smoking durations.

## Materials and methods

### Patients

This retrospective study was approved by the institutional ethics committee of our hospital, and the need for informed consent was waived. CCTA images of type 2 DM patients acquired between January 2018 and December 2020 in our hospital were reviewed. The inclusion criteria were as follows: clinical diagnosis of type 2 DM; patients with complete clinical data; and patients with coronary artery plaques on CCTA. The exclusion criteria were as follows: the image quality of CCTA was too poor to identify the state of the coronary artery; patients with a history of percutaneous coronary intervention or coronary artery bypass grafting; and patients with growth diseases, concomitant neuroendocrine tumors, severe renal failure (estimated glomerular filtration rate < 30 mL/min/1.73 m^2^), severe liver failure. Consequently, 1058 patients were enrolled in our study. The clinical data of the patients consisted of age, sex, body mass index (BMI), duration of DM, presence of complications of DM, duration of smoking, hypertension history, dyslipidemia history, statins use, diabetes treatment, systolic blood pressure, and diastolic blood pressure. The laboratory data included fasting blood glucose, HbA1c, triglycerides, cholesterol, high-density lipoprotein cholesterol (HDL-C), and low-density lipoprotein cholesterol (LDL-C).

### CCTA Scanning Protocols

CCTA scanning of all the patients was performed by using a Revolution CT scanner (GE Healthcare, Waukesha, WI, USA) or a Siemens dual-source CT scanner (SOMATOM Definition, Siemens Medical Solutions, Forchheim, Germany). Beta-blocker was not used for the heart rate reduction. Patients were examined in the supine position. A 70-90 ml (tailored to body weight) bolus of iodinated contrast agent (iopamidol, 370 mg of iodine/ml; Bracco, Shanghai, China) was injected into an antecubital vein at a flow rate of 5 ml/s followed by a 30 ml saline flush at the same flow rate. The coverage of the CT scan was from the tracheal bifurcation to 20 mm below the inferior cardiac apex. For the Revolution CT system, the tube voltage and tube current were set automatically by kV Assist and Smart-mA based on the scout image of the patients, collimation was 256×0.625 mm, and the rotation time was 0.28 seconds. For the SOMATOM Definition system, the tube voltage was 100~120 kV, the tube current was 220 mAs, the collimation was 64/128×0.6 mm, and the rotation time was 0.33 s. Retrospective electrocardiographic gating was used to eliminate artifacts of cardiac motion. Subsequently, the initial data set was reconstructed upon completion of the scan, and images were transferred to image-processing workstations (AW volumeshare5, GE Healthcare, Waukesha, WI, USA; or Syngo-Imaging, Siemens Medical Solution Systems, Forchheim, Germany) for image analysis. Alternative image reconstruction methods, including multiplanar reconstruction, maximum intensity projection, volume reconstruction, and curvature plane reconstruction, were used for the evaluation of coronary artery plaques.

### Image Analysis

**Figure 1:**
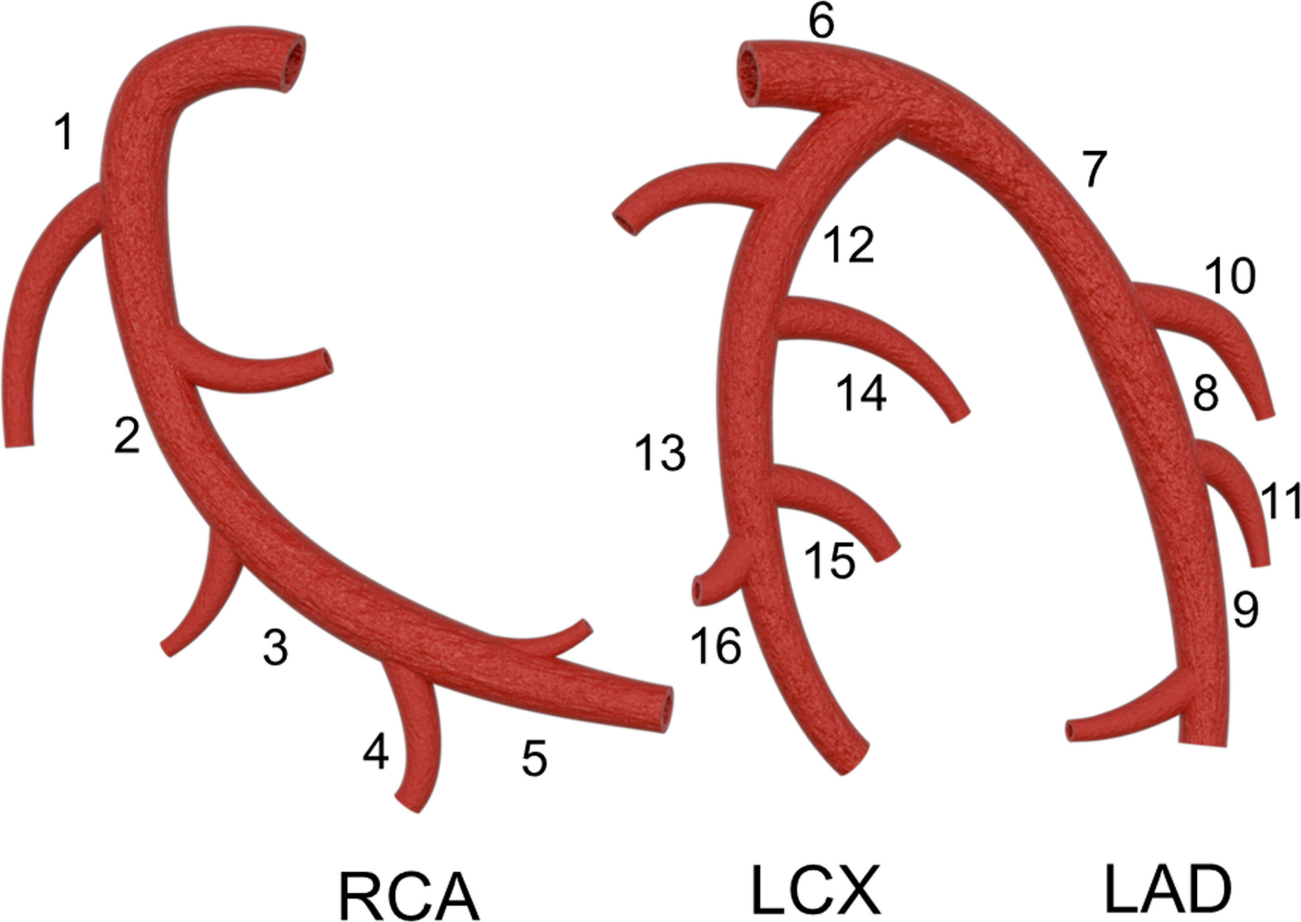
Coronary artery segments: 1 proximal segment of right coronary artery (RCA); 2 middle segment of RCA; 3 distal segment of RCA; 4 right posterolateral artery; 5 posterior descending artery; 6 left main coronary artery; 7 proximal segment of left anterior descending artery LAD; 8 middle segment of LAD; 9 distal segment of LAD; 10 first diagonal branch; 11 second diagonal branch; 12 proximal segment of left circumflex (LCX); 13 distal segment of LCX; 14 first obtuse marginal branch; 15 second obtuse marginal branch; 16 left posterolateral artery.

**Figure 2:**
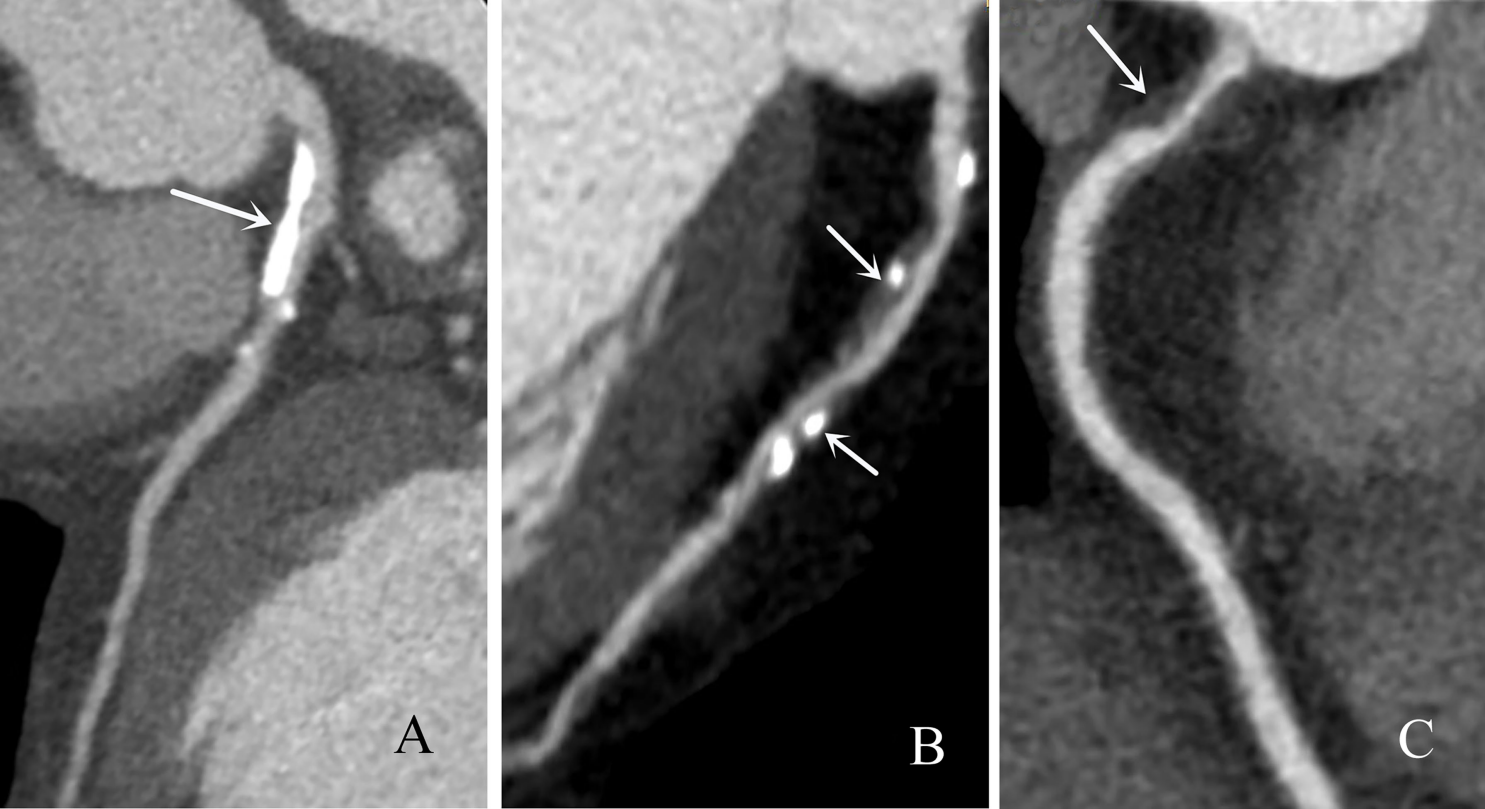
Representative CCTA images of types of coronary artery plaques. **(A)** Calcified plaques (arrow), **(B)** mixed plaque (arrow) and **(C)** noncalcified plaque (arrow).

Two cardiovascular radiologists blinded to the clinical information of the patients analyzed the images independently. The two observers reached a consensus by discussion when there were disagreements. In this study, coronary arteries were divided into 4 branches, including the left main (LM), left anterior descending (LAD), left circumflex (LCX), and right coronary artery (RCA), and into 16 segments according to a modified standard of the American Heart Association (**Figure 1**) (12, 13). Plaques were visually classified as calcified plaques (plaques with higher CT attenuation than contrast-enhanced lumen, **Figure 2A**), noncalcified plaques (plaques with lower CT density than contrast-enhanced lumen without any calcification, **Figure 2B**), and mixed plaque (calcification accompanied by noncalcified elements in a single plaque, **Figure 2C**). The extent of stenosis caused by plaques was classified as obstructive and nonobstructive based on a threshold of 50% luminal narrowing. The segment involvement score (SIS) was defined as the total number of coronary artery segments with plaques (ranging from 0-16), reflecting the extent of the plaques. The segment stenosis score (SSS) was defined as the sum of the extent grades based on CAD-RADS (14) (grade 0, no visible luminal stenosis; grade 1, < 25% luminal stenosis; grade 2, 25–49% luminal stenosis; grade 3, 50–69% luminal stenosis; grade 4, 70–99% luminal stenosis; and grade 5, totally occluded) of all segments (ranging from 0 to 80) and may reflect the severity of stenosis.

### Statistical Analysis

Statistical analysis was performed using SPSS software (version 25.0). Baseline clinical data were stratified based on smoking history (whether there was a history of smoking). Baseline clinical and laboratory data, the number of involved coronary vessels and segments, the number and types of plaques, and the extent of luminal narrowing caused by plaques were analyzed statistically in each patient. Categorical variables are expressed as numbers and percentages, and continuous variables are expressed as the mean ± standard deviation. The chi-square test was used for categorical variables. Multivariate logistic regression was used to analyze the associations of plaque characteristics between the nonsmoker and smoker groups. Continuous variables with normal distributions were compared using Student’s *t-*test. Continuous variables with nonnormal distributions were compared using the Wilcoxon rank-sum test. The associations of the mean number of diseased vessels, SIS, and SSS with the subgroups mentioned before were analyzed. The smoker group was further divided into three subgroups according to the duration of smoking (≤20 years, 20~40 years, and > 40 years) to examine the effect of smoking duration on the coronary artery plaque type and burden. A two-tailed P value of less than 0.05 was considered to indicate a significant difference.

## Results

### Study Population

A total of 1058 patients with DM who were found to have coronary artery plaques on CCTA were evaluated. The baseline clinical information is shown in **Table 1**. Smoking was defined as current or previous smoking of at least one cigarette per day for at least one year (15). According to the smoking history, the patients were divided into smoker and nonsmoker groups (with and without a smoking history, respectively). There were 610 patients in the nonsmoker group and 448 patients in the smoker group. Patients in the smoker group were more likely to be male and younger, have a lower prevalence of hypertension, a shorter duration of DM, a higher level of total cholesterol and LDL-C, and a lower level of HDL-C than those in the nonsmoker group (all P-values < 0.05). BMI, systolic blood pressure, diastolic blood pressure, fasting blood glucose, HbA1c, triglycerides, dyslipidemia, statins use, and complications of DM patients were not significantly different between the smoker and nonsmoker groups (all P-values > 0.05).

**Table 1:** Baseline characteristics of DM patients with coronary artery plaques.

Characteristics	All patients (n = 1058)	Nonsmoker (n = 610)	Smoker (n = 448)	P-value
Age (years)	69.8 ± 10.3	71.8 ± 9.5	67.2 ± 10.7	<0.001
Male	689 (65.1%)	256 (42.0%)	433 (96.7%)	<0.001
Body mass index (kg/m^2^)	24.8 ± 3.2	24.8 ± 3.3	24.8 ± 3.1	0.920
Duration of DM (year)	10 ± 8	10 ± 8	9 ± 8	0.012
Duration of smoking (year)	─	─	32 ± 14	─
Hypertension	834 (78.8%)	498 (81.6%)	336 (75.8%)	0.009
Dyslipidemia	331 (31.3%)	192 (31.5%)	139 (31.0%)	0.876
Statins use	299 (28.3%)	175 (28.7%)	124 (27.7%)	0.718
Fasting blood glucose (mmol/L)	7.60 ± 2.43	7.57 ± 2.46	7.64 ± 2.38	0.641
HbA1c (%)	7.56 ± 1.56	7.49 ± 1.47	7.66 ± 1.67	0.383
Triglyceride (mmol/L)	1.71 ± 1.21	1.75 ± 1.31	1.66 ± 1.06	0.480
Total cholesterol (mmol/L)	3.91 ± 1.16	3.87 ± 1.18	3.96 ± 1.13	0.030
HDL-C (mmol/L)	1.13 ± 0.32	1.14 ± 0.32	1.11 ± 0.32	0.028
LDL-C (mmol/L)	2.19 ± 0.95	2.13 ± 0.94	2.28 ± 0.95	0.001
Systolic BP (mmHg)	139 ± 20	139 ± 19	140 ± 21	0.408
Diastolic BP (mmHg)	80 ± 14	79 ± 14	80 ± 14	0.084
Diabetes treatment				
Oral, n (%)	763 (72.1%)	445 (74.6%)	318 (71.0%)	0.191
Insulin, n (%)	326 (30.8%)	188 (30.8%)	138 (30.8%)	0.996
Complications of diabetes				
Diabetic nephropathy	96 (9.1%)	56 (9.2%)	40 (8.9%)	0.888
Diabetic retinopathy	70 (6.6%)	40 (6.6%)	30 (6.7%)	0.928
Peripheral neuropathy	145 (13.7%)	73 (12.0%)	72 (16.1%)	0.383

Data are expressed as means ± SD, or numbers (%).

DM, diabetes mellitus; HDL-C, high-density lipoprotein cholesterol; LDL-C, low-density lipoprotein cholesterol; BP, blood pressure.

### The Anatomical Distribution of Coronary Artery Plaques in DM Patients

**Figure 3:**
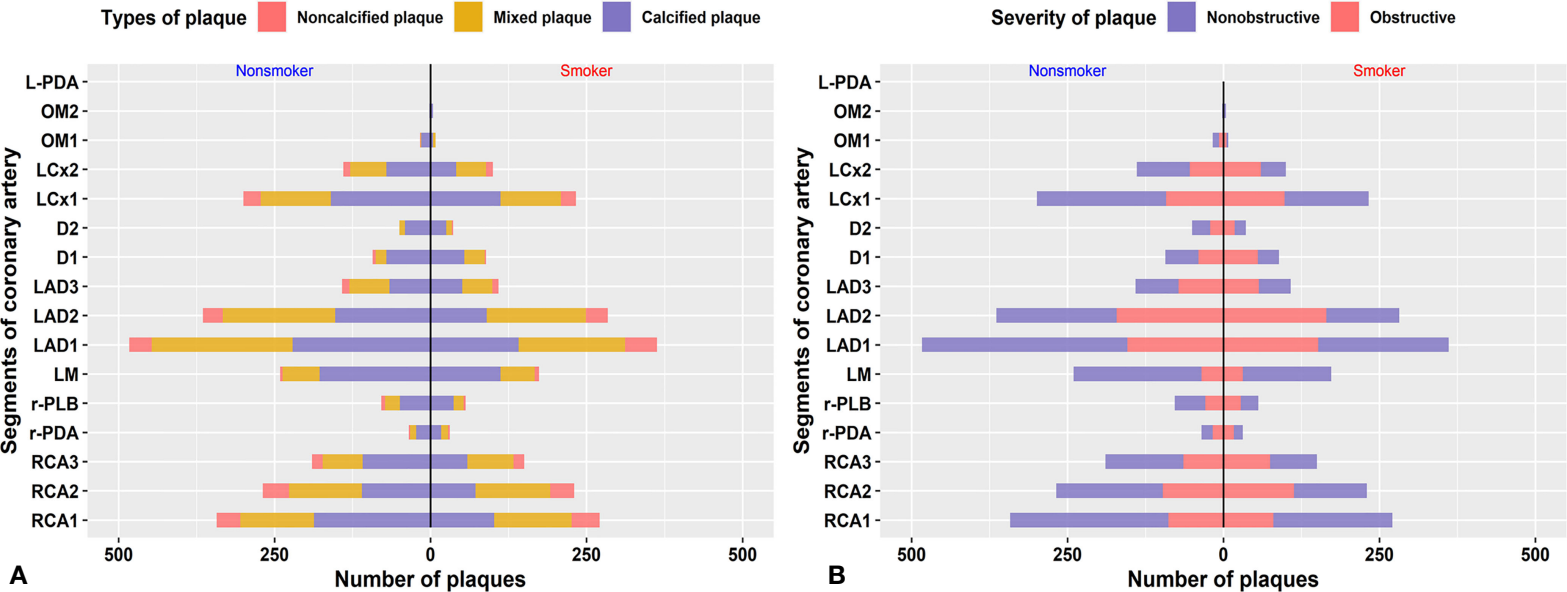
Anatomical distribution of plaques. Distribution of different types of plaque **(A)** and grade of stenosis **(B)** caused by plaque in each segment of the coronary artery.

In total, 1181 diseased coronary vessels with 2128 diseased segments and 1563 diseased coronary vessels with 2738 diseased segments were found in the smoker and nonsmoker groups, respectively. The anatomical distribution of the plaques is shown in **Figure 3**. In both the smoker and nonsmoker groups, the most frequently diseased coronary vessel was the LAD artery [417 vessels (35.3%) and 558 vessels (35.7%), respectively], followed by the RCA [337 vessels, 28.5%; and 428 vessels, 27.4%, respectively], LCX [254 vessels (21.5%) and 337 vessels (21.6%), respectively] and LM [173 vessels (14.7%) and 240 vessels (15.3%), respectively]. The three most common involved coronary artery segments in both groups were the proximal and middle segments of the LAD artery and the proximal RCA [smoker: 359 segments (16.9%), 283 segments (13.3%) and 270 segments (12.7%), respectively; nonsmoker: 483 segments (17.6%), 366 segments (13.4%) and 340 segments (12.4%), respectively], consecutively.

### Comparison of the Types of Coronary Artery Plaques and Grade of Stenosis Between DM Patients With and Without a Smoking History

A total of 4870 coronary plaques were analyzed. The types of coronary artery plaque and coronary artery stenosis stratified by smoking history are shown in **Table 2**. DM patients in the smoker group had more mixed plaques and noncalcified plaques than those in the nonsmoker group (2.2 ± 2.5 *vs.* 1.7 ± 2.1 and 0.6 ± 1.0 *vs.* 0.4 ± 0.9 per patient, respectively; both P-values < 0.05). Mixed plaques and noncalcified plaques were more common, and calcified plaques were less frequently observed in the smoker group than in the nonsmoker group (mixed: 45.5% *vs.* 38.6%, noncalcified: 11.6% *vs.* 8.6% and calcified: 42.9% *vs.* 52.8%; all P-values < 0.001). DM patients in the smoker group had more obstructive plaques than those in the nonsmoker group (2.1 ± 2.7 *vs.* 1.5 ± 2.5 per patient, P < 0.001). Regarding the grade of luminal stenosis, obstructive plaques were more frequently observed in the smoker group than in the nonsmoker group (44.8% *vs.* 34.4%; P < 0.001).

**Table 2:** Characteristics of coronary artery plaque detected by CCTA in DM patients with and without a smoking history.

Characteristics	All patients (n = 1058)	Nonsmoker (n = 610)	Smoker (n = 448)	P-value
**Number of plaques**	4870	2741	2129	
**Plaque burden**				
Calcified plaque	2.2 ± 2.4	2.4 ± 2.4	2.0 ± 2.3	0.010
Mixed plaque	1.9 ± 2.3	1.7 ± 2.1	2.2 ± 2.5	0.007
Noncalcified plaque	0.5 ± 0.9	0.4 ± 0.9	0.6 ± 1.0	0.001
Nonobstructive plaque	2.8 ± 2.2	3.0 ± 2.2	2.6 ± 2.1	0.018
Obstructive plaque	1.8 ± 2.6	1.5 ± 2.5	2.1 ± 2.7	<0.001
Diseased vessels	2.6 ± 1.0	2.6 ± 1.1	2.6 ± 1.0	0.234
SIS	4.6 ± 2.9	4.5 ± 3.0	4.8 ± 2.9	0.069
SSS	10.3 ± 9.4	9.7 ± 9.1	11.1 ± 9.7	0.005
**Plaque type**				
Calcified plaque	2361 (48.5%)	1447 (52.8%)	914 (42.9%)	<0.001
Mixed plaque	2027 (41.6%)	1059 (38.6%)	968 (45.5%)	<0.001
Noncalcified plaque	482 (9.9%)	235 (8.6%)	247 (11.6%)	<0.001
**Stenosis of coronary artery**				<0.001
Nonobstructive stenosis	2974 (61.1%)	1799 (65.6%)	1175 (55.2%)	
Obstructive stenosis	1896 (38.9%)	942 (34.4%)	954 (44.8%)	
**Number of single/multiple vessel disease**				0.275
Single	198 (18.7%)	121 (19.8%)	77 (17.2%)	
Multiple	860 (81.3%)	489 (80.2%)	371 (82.8%)	

DM, diabetes mellitus; SIS, segment involvement score; SSS, segment stenosis score. Data are expressed as means ± SD or numbers (%).

### Comparison of the Extent of Coronary Artery Plaques Between DM Patients With and Without a Smoking History

The extents of coronary artery plaque and coronary artery stenosis stratified by smoking history are shown in **Table 2**. DM patients in the smoker group had a higher SSS than those in the nonsmoker group (11.1 ± 9.7 *vs.* 9.7 ± 9.1, P < 0.05). There was no significant difference in multivessel disease, coronary vessels, or SIS between the two groups (all P-values>0.05).

### Multivariate Regression Analysis of Smoking History With Coronary Artery Plaque Presence, Extent, and Severity

**Figure 4:**
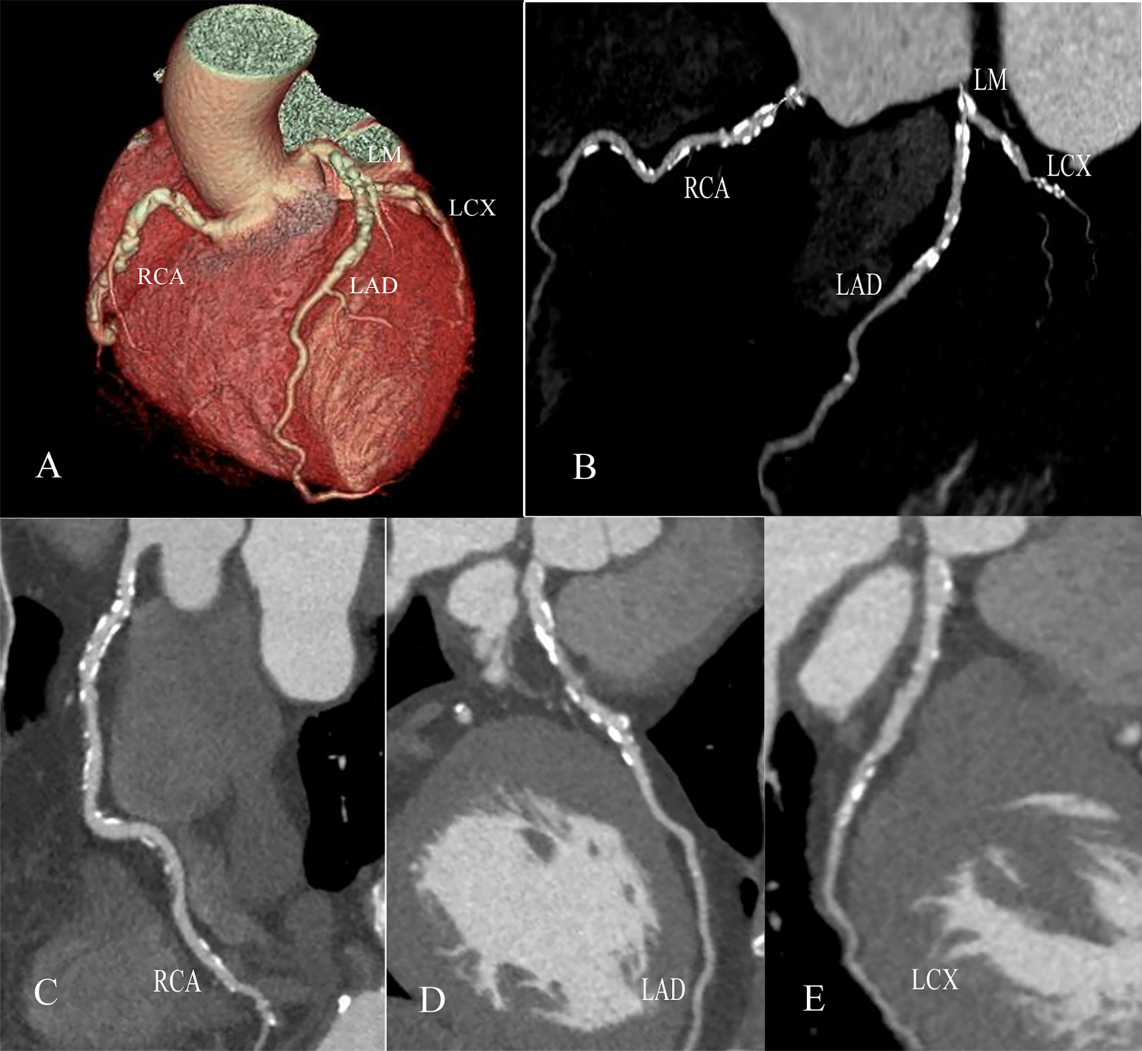
Multivessel disease in a male with type 2 diabetes mellitus with a 40-year smoking history. Volume rendering image **(A)**, two-dimensional CT image **(B)** and curvature plane reconstruction images **(C–E)** show the nonsmooth edges and diffuse calcified and mixed plaques of the LM, LAD, RCA and LCX.

As shown in **Table 3**, multivariate regression analysis was performed to control for age, sex, BMI, duration of DM, hypertension, dyslipidemia, total cholesterol, HDL-C and LDL-C. Compared to the nonsmoker group, smokers demonstrated an increased odds ratio (OR) of any noncalcified plaque [OR= 1.423 (95% CI, 1.074-1.887); P=0.014), obstructive plaque [OR=1.884 (1.458-2.436); P<0.001], multivessel disease [OR=1.491 (1.066-2.085); P=0.020] (**Figure 4**), SIS≥4 [OR=1.662 (1.278-2.162); P<0.001] and SSS≥7 [OR=1.562 (1.204-2.025); P=0.001].

**Table 3:** Multivariate regression analysis of the association of coronary artery plaques in diabetes with smoking history.

Dependent variables	Odds ratio (95% CI)	P-value
Any calcified plaque	1.098 (0.772-1.560)	0.604
Any mixed plaque	1.158 (0.843-1.592)	0.365
Any noncalcified plaque	1.423 (1.074-1.887)	0.014
Any nonobstructive plaque	0.788 (0.441-1.406)	0.419
Any obstructive plaque	1.884 (1.458-2.436)	<0.001
Any multiple vessel disease	1.491 (1.066-2.085)	0.020
Segment involvement score ≥ 4	1.662 (1.278-2.162)	<0.001
Segment stenosis score ≥ 7	1.562 (1.204-2.025)	0.001

CI, confidence interval. All odds ratios were obtained by binary logistic regression adjusted for age, sex, duration of diabetes mellitus, hypertension, dyslipidemia, body mass index, total cholesterol, high-density lipoprotein cholesterol and low-density lipoprotein cholesterol.

### Multivariate Regression Analysis for the Duration of Smoking Associated With Coronary Artery Disease

**Figure 5:**
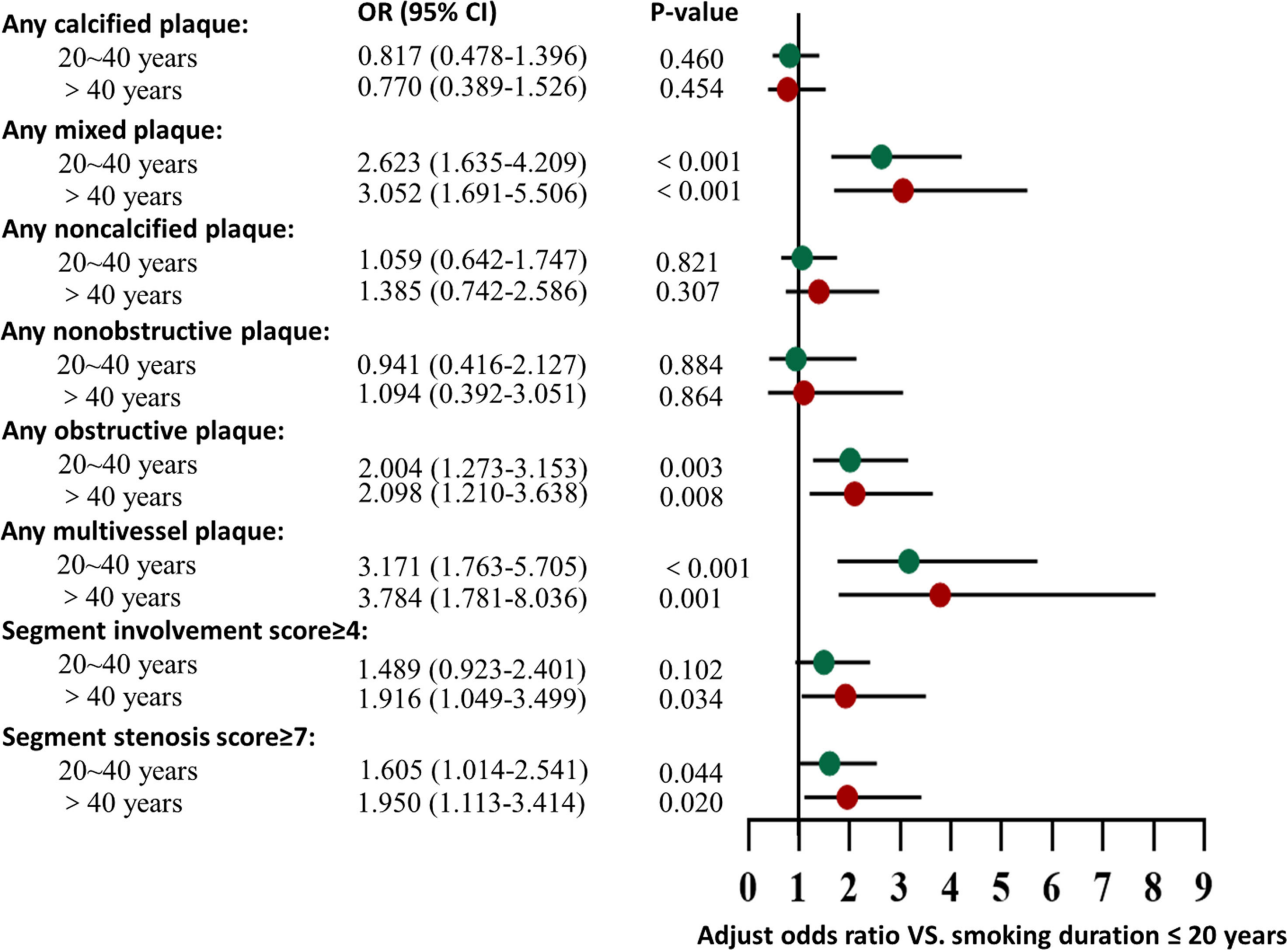
Association of smoking duration with CCTA measures. Adjusted odds ratio of all types of plaque, severity, and extent by comparison to DM patients with a smoking history ≤20 years, among DM patients with a smoking history of 20~40 years and > 40 years. The multivariate regression analysis was adjusted for age, sex, BMI, duration of DM, hypertension, dyslipidemia, total cholesterol, high-density lipoprotein cholesterol and low-density lipoprotein cholesterol.

As shown in **Table 4** and **Figure 5**, compared to DM patients with a smoking duration ≤20 years, smoking durations of 20 to 40 years and >40 years had more mixed plaques [OR=2.623 (1.635-4.209) and 3.052 (1.691-5.506), respectively; both P-values<0.001], obstructive plaques [OR=2.004 (1.273-3.153) and 2.098 (1.210-3.638), P=0.003 and 0.008, respectively], multivessel disease [OR=3.171 (1.763-5.705) and 3.784 (1.781-8.036), P<0.001 and P=0.001, respectively] and SSSs≥7 [OR=1.605 (1.014-2.541) and 1.950 (1.113-3.414), P=0.044 and 0.020, respectively]. DM patients with a smoking duration >40 years had more SISs≥4 [OR=1.916 (1.049-3.499), P=0.034]. Smoking duration was not an independent risk factor for calcified plaques and noncalcified plaques according to the multivariate regression analysis.

**Table 4:** Multivariate regression analysis of the association of coronary artery plaques in diabetes with smoking duration.

Duration of smoking (years)	≤20 years (n=115)	20~40 years (n = 233)		>40 years (n = 100)	
		OR (95% CI)	P-value	OR (95% CI)	P-value
	Reference				
Any calcified plaque		0.817 (0.478-1.396)	0.460	0.770 (0.389-1.526)	0.454
Any mixed plaque		2.623 (1.635-4.209)	< 0.001	3.052 (1.691-5.506)	<0.001
Any noncalcified plaque		1.059 (0.642-1.747)	0.821	1.385 (0.742-2.586)	0.307
Any nonobstructive plaque		0.941 (0.416-2.127)	0.884	1.094 (0.392-3.051)	0.864
Any obstructive plaque		2.004 (1.273-3.153)	0.003	2.098 (1.210-3.638)	0.008
Multivessel disease		3.171 (1.763-5.705)	< 0.001	3.784 (1.781-8.036)	0.001
Segment involvement score ≥4		1.489 (0.923-2.401)	0.102	1.916 (1.049-3.499)	0.034
Segment stenosis score ≥ 7		1.605 (1.014-2.541)	0.044	1.950 (1.113-3.414)	0.020

OR, odds ratio; CI, confidence interval. All ORs were obtained by multivariate logistic regression adjusted for age, sex, duration of diabetes mellitus, hypertension, dyslipidemia, body mass index, total cholesterol, high-density lipoprotein cholesterol and low-density lipoprotein cholesterol.

## Discussion

The present study had four main findings. First, compared to those in the nonsmoker group, DM patients in the smoker group showed a higher prevalence of mixed plaques and noncalcified plaques and a lower prevalence of calcified plaques. Second, compared to those in the nonsmoker group, DM patients in the smoker group showed a higher prevalence of obstructive plaques. Third, smoking was an independent risk factor for noncalcified plaques, obstructive coronary artery disease, and more extensive lesions in DM patients after adjustment for confounding factors. Fourth, DM patients with longer smoking durations had a higher likelihood of mixed plaques and obstructive coronary artery disease and more extensive lesions than those with shorter smoking durations.

DM patients already have a high coronary plaque burden, which indicates active progression of atherosclerosis (9, 16), and smoking promotes inflammation and leads to longer-term adverse cardiac outcomes (17). Possible explanations for the formation of plaques could be that smoking affects each stage of the atherosclerotic process through the activity of the chemical constituents of cigarette smoke. The high oxidant and inflammatory properties of cigarette smoke can directly damage the endothelium of arteries, aggravate the inflammatory response, promote vascular remodeling, and lead to plaque destabilization (18). Decreased nitric oxide bioavailability, increased expression of adhesion molecules and subsequent endothelial dysfunction lead to smoking-induced vascular dysfunction (19, 20). The increasing adherence of platelets and macrophages induced by smoking contributes to an inflammatory environment (19), and the presence of macrophage infiltration at culprit lesions is associated with more vulnerable plaque features as well as a worse prognosis (21).

For the types of plaques, the present data showed that the proportion of mixed plaques and noncalcified plaques was higher and that of calcified plaques was lower in the smoker group than in the nonsmoker group; when adjusted for confounding factors, smoking was independently associated with an increased adjusted odds ratio of the presence of noncalcified plaques, and a longer smoking duration was correlated with mixed plaques. Noncalcified plaques and mixed plaques show relatively high incidences of major adverse cardiovascular events, and noncalcified plaque is associated with an increased risk of atherosclerotic cardiovascular disease (22, 23). Similar to our results, a previous study of tobacco use with coronary atherosclerosis demonstrated the association of smoking with the presence of all coronary plaque types, which reflected a heavier coronary plaque burden of smokers (11). The explanation for a lower proportion of calcified plaques in the smoker group than the nonsmoker group could be that smoking may aggravate the inflammatory activity and result in unstable atherosclerotic plaque. In addition, this difference also could be attributed to the differences in the baseline characteristics as there was no significant difference of any calcified plaques between smoker and nonsmoker groups when adjusted for confounding factors.

It has been reported that the percent of low-density plaque content and mild calcification could predict late plaque events in asymptomatic patients with DM, and lumen narrowing caused by plaques increases the probability of an acute event (24). Calcified plaques, which are more likely to be stable plaques (25), can also lead to severe coronary artery stenosis. As shown in our study, cigarette smoke and longer smoking duration were independently associated with obstructive stenosis caused by coronary artery plaques. This result reminds us of the importance of CCTA evaluation because the prognosis of DM patients with obstructive coronary artery disease was worse than that of DM patients with the nonobstructive disease over a follow-up period of more than 5 years (26).

According to the present data, DM patients with a smoking history had a higher risk of more extensive coronary artery disease than nonsmokers after adjustment for confounding factors. Patients with extensive coronary artery disease can benefit from surgical revascularization. The evaluation of the extent of coronary artery plaque assessment could be important for treatment. For DM patients with three-vessel coronary artery disease, coronary artery bypass grafting results in better long-term survival than percutaneous coronary intervention (27). A study also indicated a comparable mortality benefit between coronary artery bypass grafting and percutaneous coronary intervention in patients with the left main disease, as the 5-year all-cause mortality in both interventions was approximately 10% (28).

A previous study indicated that with increasing smoking duration, the adjusted hazard ratio for coronary heart disease also increased (3). It has also been proven that cumulative exposure to smoking is a significant risk factor for atherosclerosis (29). Furthermore, there is a dose-response relationship between smoking duration and coronary heart disease (3). Discoveries have shown a higher risk of coronary heart disease in patients with longer smoking duration, even with a lower smoking intensity (30, 31). Another study revealed that patients with longer pack-year exposure (≥ 12 years) were more likely to have more extensive plaques on CCTA examination than never-smokers (11). Similar to previous studies, our data of DM patients showed that a longer duration of smoking (20 to 40 years and > 40 years) was independently associated with a higher prevalence of more extensive lesions (multivessel disease and SSS≥7) compared with a lower duration of smoking (≤20 years, all P-values <0.05). In contrast to those of DM patients with a smoking duration of 20 to 40 years, the ORs of multivessel disease, SIS≥4, and SSS≥7 were even higher in DM patients with smoking duration longer than 40 years.

There are some limitations of our research. First, since this is a single-center study, selection bias is inevitable in this study. Therefore, the results of this study remain to be verified by multicenter studies. Second, traditional coronary angiography results of the extent of coronary stenosis were not correlated with our CCTA findings, as CCTA can distinguish different types of plaques, and the assessment of coronary plaques by CCTA has been widely accepted. Third, as a retrospective study, the evolution of the disease remains to be discussed in further follow-up studies. Finally, nondiabetic patients were not enrolled in our study. Thus, further exploration is required to investigate the potential difference in characteristics of coronary artery disease between patients with and without DM.

## Conclusion

CCTA detected a heavy plaque burden in DM patients. Smoking is independently associated with the presence of noncalcified plaques, obstructive plaques, and more extensive coronary artery plaques in DM patients, and a longer smoking duration is significantly associated with a higher risk of mixed plaques, obstructive plaques, and more extensive coronary artery plaques than a shorter smoking duration.

## Data Availability Statement

The datasets presented in this article are not readily available because of the privacy of patient information. Requests to access the datasets should be directed to Z-GY, yangzg666@163.com.

## Ethics Statement

The studies involving human participants were reviewed and approved by the Biomedical Research Ethics Committee, West China Hospital of Sichuan University (Chengdu, Sichuan, China). Written informed consent for participation was not required for this study in accordance with the national legislation and the institutional requirements.

## Author Contributions

YJ, TP, YL, and Z-GY contributed to the conception and design of the study. YJ, TP, W-LQ, and W-FY collected the data. YJ, TP, and RS participated in the data analysis and interpretation. YJ wrote the manuscript with contributions from all authors. All authors contributed to the article and approved the submitted version.

## Funding

This study was financially supported by the 1·3·5 project for disciplines of excellence of West China Hospital, Sichuan University (ZYGD18013).

## Conflict of Interest

The authors declare that the research was conducted in the absence of any commercial or financial relationships that could be construed as a potential conflict of interest.

## Publisher’s Note

All claims expressed in this article are solely those of the authors and do not necessarily represent those of their affiliated organizations, or those of the publisher, the editors and the reviewers. Any product that may be evaluated in this article, or claim that may be made by its manufacturer, is not guaranteed or endorsed by the publisher.

## Abbreviations

DM, diabetes mellitus; CCTA, coronary computed tomography angiography; BMI, body mass index; HDL-C, high-density lipoprotein cholesterol; LDL-C, low-density lipoprotein cholesterol; RCA, right coronary artery; LM, left main; LAD, left anterior descending; LCX, left circumflex; SIS, segment involvement score; SSS, segment stenosis scores; OR, odds ratio.
